# Aprocitentan in resistant hypertension with chronic kidney disease: balancing albuminuria reduction against early volume risk

**DOI:** 10.1038/s41371-026-01163-4

**Published:** 2026-06-02

**Authors:** Lucas Maciel de Almeida Corrêa, Yan Roberth Delmiro Silva, Juliana Zanella, Gabriel Costa de Santana

**Affiliations:** 1 Faculdade de Medicina de São José do Rio Preto (FAMERP), São José do Rio Preto, Brazil; 2 Universidade Federal de Alagoas (UFAL), Arapiraca, Brazil; 3 Universidade Federal de Santa Catarina (UFSC), Florianópolis, Brazil; 4 Universidade Salvador (UNIFACS), Salvador, Brazil

**Keywords:** Renovascular hypertension, Chronic kidney disease

## Abstract

Resistant hypertension in chronic kidney disease (CKD) remains a high-risk clinical phenotype associated with poor blood pressure (BP) control, accelerated kidney disease progression, and excess cardiovascular burden. Aprocitentan, a dual endothelin receptor antagonist approved as add-on therapy for hypertension, offers a new option for patients with resistant hypertension, but its incorporation into CKD care remains constrained by persistent concerns regarding fluid retention. This review synthesizes data from the phase 3 PRECISION program and related endothelin literature to examine how BP lowering, albuminuria reduction, and early volume-related adverse effects should be interpreted in patients with resistant hypertension and CKD. Available evidence suggests that aprocitentan provides clinically meaningful and sustained antihypertensive effects while also producing marked reductions in albuminuria in CKD-enriched high-risk phenotypes, with signals supporting at least partial dissociation between antiproteinuric benefit and systemic BP lowering. The major trade-off is a predictable, front-loaded volume signal characterized by early edema, weight gain, and hemodilution, which appears most relevant during the first weeks after treatment initiation and may be manageable with proactive sodium and diuretic strategies. We also discuss the physiological rationale for combining endothelin blockade with SGLT2 inhibitors as a potential strategy to improve the balance between efficacy and tolerability. Overall, aprocitentan should be interpreted within a clinical benefit-risk framework in which resistant hypertension is the primary therapeutic target, while albuminuria reduction and early volume surveillance define its potential role in CKD.

## Resistant hypertension in ckd: the re-emergence of endothelin blockade

Resistant hypertension (RHT), defined as blood pressure that remains above goal despite treatment with 3 antihypertensive drugs of different classes at maximally tolerated doses, ideally including a diuretic, or as blood pressure controlled only with ≥4 agents after exclusion of pseudoresistance, remains a high-risk phenotype associated with accelerated cardiovascular and kidney complications, and is particularly prevalent in patients with chronic kidney disease (CKD), where sodium retention, sympathetic activation, and neurohormonal dysregulation converge to blunt the response to conventional multidrug therapy [1–3]. Against this backdrop, endothelin-1 (ET-1) has re-emerged as a clinically actionable node: ET-1 is a potent vasoconstrictor and pro-fibrotic mediator that amplifies vascular tone, inflammation, and glomerular injury, and its activity is upregulated in CKD and has been implicated in treatment-resistant hypertension [4, 5]. Aprocitentan is an oral, dual endothelin receptor antagonist (ETA/ETB) developed as add-on therapy for difficult-to-control and resistant hypertension, with a pharmacologic profile designed to provide sustained blood pressure (BP) lowering while minimizing off-target liabilities [4, 6, 7]. In March 2024, aprocitentan received its first approval in the United States for the treatment of hypertension, in combination with other antihypertensive drugs, in adults not adequately controlled on other drugs [8]. This regulatory milestone was largely informed by the phase 3 PRECISION program, which evaluated aprocitentan in patients with resistant hypertension [9]. More importantly, aprocitentan should be viewed as the latest attempt to solve a longstanding endothelin paradox: substantial antiproteinuric and antihypertensive potential on one side, and fluid-retention liability on the other. Prior ERA programs showed that the key question is not whether the class can reduce albuminuria, but whether efficacy can be separated from edema and heart-failure risk through dose selection, patient selection, and early surveillance [10, 11]. Although prior experience with other endothelin receptor antagonists helps contextualize both antiproteinuric potential and fluid-retention liability, this review focuses on aprocitentan because its dual ETA/ETB profile and resistant-hypertension indication create a distinct clinical positioning that should not be considered interchangeable with earlier class programs [4, 9–12].

## Strategy and selection of evidence

This narrative review was informed by a focused literature search performed in PubMed/MEDLINE, Scopus, Embase, and Web of Science using the terms “aprocitentan”, “chronic kidney disease”, and “resistant hypertension”. Priority was given to randomized clinical trials, mechanistic studies, clinically relevant reviews, and key translational reports directly informing the efficacy and safety profile of aprocitentan in resistant hypertension and CKD. Reference lists of selected articles were also screened to identify additional relevant publications. This review was designed as a focused clinical synthesis centered on aprocitentan in resistant hypertension with CKD, rather than a comprehensive class-wide review of endothelin receptor antagonists.

## The kidney signal within a resistant hypertension framework: albuminuria reduction beyond bp lowering

Beyond BP reduction, an important rationale for targeting the endothelin axis in resistant hypertension with CKD is the possibility of kidney protection beyond BP lowering. ET-1 signaling promotes podocyte dysfunction, mesangial activation, and intrarenal inflammation, pathways that are tightly linked to albuminuria and progressive nephron loss [5]. Accordingly, a clinically meaningful and reproducible reduction in urine albumin-to-creatinine ratio (UACR) could represent a biologically plausible and trial-supported surrogate of kidney benefit in albuminuric CKD, particularly when the magnitude of reduction exceeds what would be expected from BP lowering alone, potentially signaling attenuation of intrarenal endothelin-driven injury [13].

This kidney-centric signal has been most clearly demonstrated in the CKD-enriched subgroup analyses from the phase 3 PRECISION program. In participants categorized as KDIGO ≥high risk (a group enriched for CKD stages 3–4 and/or significant albuminuria), aprocitentan produced marked early UACR reductions at week 4: −47.1% (95% CI, −56.1 to −36.4) with 12.5 mg and −59.6% ( − 69.1 to −47.1) with 25 mg, versus −2.4% ( − 24.4–26.1) with placebo [9, 14]. At week 36 (after 32 weeks of aprocitentan 25 mg), UACR reduction persisted ( − 61.6% [−70.2 to −50.7]), and during double-blind withdrawal, UACR rebounded in those switched to placebo (108.3% [46.0–197.1]) while remaining stable or further improving in those continuing aprocitentan ( − 12.9% [−26.1–2.7]) [14]. Importantly, correlations between changes in unattended systolic BP and UACR were modest (R ≈ 0.12–0.32), supporting at least partial dissociation between the antiproteinuric effect and BP lowering [14]. Short-term kidney function changes were small and directionally consistent with hemodynamic shifts (mean eGFR change from baseline to week 4: 0.5, −2.5, and −0.4 mL/min/1.73 m² for 12.5 mg, 25 mg, and placebo, respectively), with minimal changes through week 48 and a chronic eGFR slope estimate of −0.9 mL/min/1.73 m²/year from weeks 6–36 on aprocitentan 25 mg [14]. At the class level, this is not merely a mechanistic hypothesis: in SONAR, selective ETA blockade with atrasentan reduced hard renal events in albuminuric diabetic CKD after an enrichment strategy designed to maximize albuminuria response while minimizing fluid-retention susceptibility, underscoring both the potential and the operational complexity of endothelin-based nephroprotection [10].

## The volume liability: edema, plasma volume expansion, and hemodilution

The major counterweight to this compelling UACR signal is fluid retention—an on-target class effect of endothelin receptor antagonists that becomes clinically salient in advanced CKD. In KDIGO ≥high-risk participants exposed to aprocitentan at any dose during PRECISION, 37.9% experienced at least one edema event, compared with 25.4% in the overall phase 3 population [14]. Notably, the risk was front-loaded: incident edema within the first 4 weeks occurred in 16.3 and 15.1% of participants randomized to aprocitentan 12.5 mg and 25 mg, respectively, versus 4.4% with placebo, and then returned to prerandomization rates by weeks 12–16 [14]. Hospitalized heart failure events were infrequent but clinically consequential; in the KDIGO ≥high-risk subgroup, 7 of 11 total heart-failure hospitalizations in the overall study occurred in this high-risk cohort, and events may have been facilitated by background diuretic de-escalation mandated by the standardized antihypertensive regimen, reinforcing the need for structured volume surveillance rather than therapeutic complacency [14]. Despite frequent edema events, permanent discontinuation attributed to edema remained uncommon in the KDIGO ≥high-risk cohort, underscoring the generally manageable nature of this adverse effect with appropriate volume control [14].

Objective markers were congruent with a predominantly volume-mediated phenotype. From baseline to week 4, estimated plasma volume increased by 14.6% (SE 2.0) and 13.7% (SE 1.4) with aprocitentan 12.5 mg and 25 mg, respectively, versus 3.2% (SE 1.5) with placebo, and reversed upon withdrawal [14]. Consistent with hemodilution, hemoglobin decreased by approximately 10 g/L at week 4 with aprocitentan ( − 10.2 [SD 8.9] and −10.3 [SD 6.8] g/L for 12.5 mg and 25 mg) compared with −2.0 (SD 7.1) g/L with placebo [14]. Mean body weight changes were modest ( ≈ 0.6–0.7 kg increase at week 4 with aprocitentan, with little change on placebo) and subsequently attenuated [14]. Mechanistic data from a randomized crossover study in healthy volunteers on high sodium intake similarly demonstrated modest, placebo-corrected weight gain ( ≈ 0.4–0.8 kg over 9 days across 10–50 mg), small hemoglobin decreases, and limited plasma volume expansion (maximum ≈5.5%) without a clear dose–response, reinforcing that aprocitentan may shift fluid distribution and hemodilution even in the absence of overt sodium retention [15].

## Distinguishing drug-related volume expansion from decompensated heart failure

In clinical practice, a central interpretive challenge is separating aprocitentan-associated edema from true cardiac decompensation. In CKD and RHT, baseline extracellular volume expansion is common and may be clinically occult; classic physiology studies demonstrate that resistant hypertension is frequently accompanied by persistent intravascular volume expansion and aldosterone excess, even under multidrug therapy [16]. Recent CKD-focused reviews emphasize that impaired natriuresis and sodium/fluid retention amplify the propensity for edema as kidney function declines, and that BP “resistance” often reflects a volume-dominant substrate rather than insufficient vasodilatory therapy [3]. Against this background, ET receptor antagonism may unmask or modestly augment pre-existing volume load, particularly early after initiation, and especially when background diuretics are reduced.

Therefore, volume expansion with aprocitentan should be conceptualized first as a pharmacodynamic signal that requires active differentiation from true decompensated heart failure, rather than being assumed to represent either benign edema or direct cardiac toxicity in every case. A pragmatic approach is to (i) document baseline “dry weight” and edema status, (ii) avoid routine down-titration of effective loop diuretic therapy when initiating standardized background regimens, (iii) intensify dietary sodium counseling, and (iv) reassess early (within 2–4 weeks) for weight gain, ankle edema, orthopnea, and objective changes such as hemoglobin dilution or rising natriuretic peptides when available [3, 14–16]. In high-risk phenotypes (advanced CKD, diabetes, prior heart failure), proactive diuretic optimization and close follow-up may allow preservation of the antiproteinuric and antihypertensive benefits while mitigating symptomatic fluid retention.

While the antiproteinuric signal in the CKD-enriched cohort is robust, these findings must be interpreted within the context of specific limitations. First, the kidney-specific data from PRECISION derive from a post hoc analysis of a CKD-enriched subgroup; although informative, the study was powered for blood pressure endpoints rather than hard renal outcomes (e.g., progression to end-stage kidney disease or doubling of serum creatinine) [9, 14]. Second, the follow-up duration in the double-blind phase (4 weeks) and the maintenance phase (32 weeks) is sufficient to detect early hemodynamic effects but remains relatively short for assessing long-term structural kidney preservation and the chronic safety profile—particularly regarding volume-mediated hemoglobin changes in a fragile CKD population [9, 14, 15]. Finally, the generalizability of these findings to non-albuminuric CKD phenotypes or to those with eGFR <15 mL/min/1.73 m² remains to be established [14].

## A clinical benefit–risk framework for resistant hypertension in CKD

**Fig. 1:**
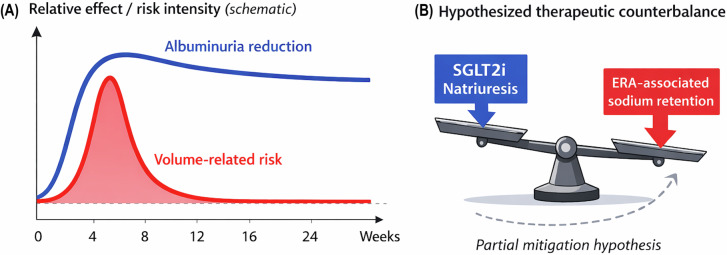
Conceptual framework of aprocitentan in resistant hypertension with chronic kidney disease. **A** Schematic representation of the temporal dissociation between albuminuria reduction (blue) and volume/edema risk (red) based on data from the PRECISION study. Note the “front-loaded” nature of the volume risk (peaking at week 4) versus the sustained antiproteinuric efficacy. **B** Mechanistic rationale for combination therapy. The addition of an SGLT2 inhibitor (natriuresis) counterbalances the sodium retention induced by the Endothelin Receptor Antagonist (ERA), potentially optimizing the therapeutic index.

A useful clinical heuristic is to frame aprocitentan’s net value in resistant hypertension with CKD as the intersection of two dose–time response curves: [1] an efficacy curve, combining BP lowering and albuminuria reduction; and [2] a volume-risk curve, in which edema and plasma volume expansion peak early and then stabilize or regress with appropriate management [14]. The apparent plateau of plasma volume expansion and incident edema between 12.5 mg and 25 mg in KDIGO ≥high-risk participants suggests that, at the subgroup level, greater antiproteinuric efficacy was not accompanied by a clearly proportional increase in measured volume markers; however, this should not be interpreted as proof of safety equivalence for individual patients [14]. Rather, individual susceptibility varies, and dose selection should be individualized, guided by early tolerability and the clinical priority placed on albuminuria reduction. This benefit–risk framework is visually summarized in Fig. 1, and the key clinical, mechanistic, and translational evidence underpinning it is synthesized in Table 1.

**Table 1:** Key clinical, mechanistic, and translational evidence informing the benefit–risk profile of aprocitentan in resistant hypertension with chronic kidney disease.

Evidence domain	Study / population	Intervention	Main efficacy signal	Main volume-related signal	Implication for benefit–risk assessment	Ref.
Direct CKD clinical signal	PRECISION post hoc CKD-enriched subgroup (KDIGO high/very high risk) with resistant hypertension	Aprocitentan 12.5 mg, aprocitentan 25 mg, or placebo on standardized background therapy	Week-4 office SBP: −13.5 and −16.6 vs −4.4 mmHg with placebo; nighttime ambulatory SBP: −9.6 and −13.8 vs −2.5 mmHg; week-4 UACR change: −47.1% and −59.6% vs −2.4%, with persistence to week 36, supporting a sustained antiproteinuric signal partly beyond BP lowering	Peripheral edema was the most common adverse event; the clinical signal is therefore best interpreted as early antiproteinuric opportunity coupled to early volume liability	Demonstrates that albuminuria reduction and blood pressure lowering emerge early in CKD, alongside a parallel early edema liability	[14]
Mechanistic aprocitentan volume biology	Randomized crossover study in healthy subjects on a high-sodium diet	Aprocitentan 10, 25, or 50 mg/day for 9 days versus placebo	No kidney efficacy endpoint; study was designed to clarify early fluid-homeostasis effects	Placebo-corrected mean body-weight gain: +0.43, +0.77, and +0.83 kg at 10, 25, and 50 mg; estimated plasma-volume increase up to 5.5%, without a clear dose-response pattern and without obvious sodium retention	Refines the interpretation of early weight gain by linking it to plasma-volume expansion rather than straightforward sodium retention alone	[15]
Class-level translational support for ERA + SGLT2 strategy	ZENITH-CKD phase 2b trial in albuminuric CKD (eGFR ≥20 mL/min/1.73 m²; UACR 150–5000 mg/g)	Zibotentan 1.5 mg + dapagliflozin 10 mg, zibotentan 0.25 mg + dapagliflozin 10 mg, or dapagliflozin + placebo	Week-12 UACR reduction versus dapagliflozin alone: −33.7% with zibotentan 1.5 mg + dapagliflozin and −27.0% with zibotentan 0.25 mg + dapagliflozin	Fluid-retention events: 18% with zibotentan 1.5 mg + dapagliflozin, 9% with zibotentan 0.25 mg + dapagliflozin, and 8% with dapagliflozin alone	Suggests that SGLT2 co-therapy may preserve antiproteinuric benefit while attenuating fluid-retention burden	[12]
Exposure- and eGFR-dependent fluid-retention analysis	Post hoc ZENITH-CKD analysis linking biomarkers, exposure, eGFR, and fluid retention	Zibotentan alone or combined with dapagliflozin across dose levels	Early changes in body weight, BNP, and hemoglobin tracked with extracellular-fluid changes, identifying clinically usable monitoring anchors	Compared with dapagliflozin alone, fluid-retention risk increased with zibotentan exposure: HR 8.50 for zibotentan 5 mg alone, 3.09 for zibotentan 5 mg + dapagliflozin, 2.70 for zibotentan 1.5 mg + dapagliflozin, and 1.21 for zibotentan 0.25 mg + dapagliflozin; lower eGFR further increased risk	Supports the use of simple clinical and laboratory markers to detect early fluid-retention risk during endothelin receptor antagonist therapy	[20]
External clinical consistency in Black patients	Preplanned PRECISION subgroup analysis in Black patients with resistant hypertension	Aprocitentan 12.5 mg, aprocitentan 25 mg, or placebo on standardized background therapy	BP reduction was similar to that observed in the overall PRECISION population, with additional reduction in albuminuria	Peripheral edema was the most frequent adverse event, with an overall tolerability profile consistent with the broader trial population	Extends the clinical applicability of aprocitentan to a particularly high-risk resistant-hypertension subgroup and supports broader external validity of the benefit–risk framework	[21]

The studies summarized here provide the evidentiary basis for interpreting aprocitentan through a benefit–risk lens in resistant hypertension with CKD. Together, they indicate that endothelin receptor antagonism may deliver clinically meaningful antiproteinuric and blood pressure effects, but that these gains are counterbalanced by an early, exposure-sensitive fluid-retention signal that warrants proactive surveillance, thoughtful dose selection, and, potentially, mitigation strategies such as SGLT2 co-therapy.

*BNP* B-type natriuretic peptide, *BP* blood pressure, *CKD* chronic kidney disease, *eGFR* estimated glomerular filtration rate, *ERA* endothelin receptor antagonist, *HR* hazard ratio, *KDIGO* kidney disease, improving global outcomes, *SBP* systolic blood pressure, *SGLT2* sodium-glucose cotransporter-2, *UACR* urine albumin-to-creatinine ratio.

## Clinical implications

Within the contemporary management of resistant hypertension, aprocitentan should be interpreted as a later-line add-on option rather than a replacement for established foundational strategies such as confirmation of true resistant hypertension, optimization of diuretic therapy, sodium restriction, and use of guideline-supported multidrug regimens, including mineralocorticoid receptor antagonism when tolerated. Its clinical relevance becomes greater in patients with CKD and persistent uncontrolled BP despite standard therapy, particularly when albuminuric risk is also present [1, 3, 9, 14].

For clinicians managing resistant hypertension in CKD, aprocitentan should be viewed not simply as another antihypertensive agent, but as a candidate add-on therapy whose appeal lies in the combination of clinically meaningful BP lowering and a potentially important antiproteinuric effect in albuminuric CKD [9, 13, 14]. The practical trade-off is an early, largely predictable volume-related signal that appears to cluster within the first 4 weeks, supporting proactive surveillance rather than reflex discontinuation of therapy [14, 15]. In carefully selected patients—especially those with resistant hypertension, albuminuric CKD, and persistent risk despite standard multidrug regimens—treatment decisions should integrate baseline volume status, sodium intake, diuretic strategy, and early follow-up for edema, weight gain, and hemodilution [3, 14–16]. Under this framework, the clinical question is less whether aprocitentan lowers BP, and more which patients can achieve a favorable balance between antihypertensive and antiproteinuric benefit and manageable early volume liability [14].

## Future directions: optimizing the therapeutic index via combination therapy

In CKD, particularly in albuminuric and higher-risk phenotypes, aprocitentan may occupy an important adjunctive niche in carefully selected patients: it couples robust blood pressure reduction with a sizable, partly BP-independent antiproteinuric effect—features that may justify its use in selected patients with resistant hypertension and albuminuric CKD, provided that early volume-related risk is actively anticipated and managed. This proposition is strengthened by broader endothelin experience: albuminuria reduction has trial-level validity as a surrogate endpoint in albuminuric CKD, and prior ETA-antagonist programs showed that kidney benefit becomes more credible when efficacy is paired with explicit safeguards against fluid retention. The trade-off is predictable early fluid retention, reflected by transient weight gain, hemodilution, and edema clustering soon after initiation; however, the CKD-enriched PRECISION subgroup supports a coherent and largely reversible safety phenotype with low discontinuation when sodium and diuretic strategies are proactive [1–3, 14–16].

Unlike highly selective ETA antagonists (e.g., zibotentan) that have primarily targeted albuminuria in albuminuric CKD, aprocitentan’s dual ETA/ETB blockade offers a distinct hemodynamic profile characterized by substantial blood pressure lowering in resistant phenotypes [4, 7, 9, 12]. Despite these mechanistic differences, fluid retention remains a shared concern [9, 12, 15]. Thus, even without direct combination trials, layering an SGLT2 inhibitor onto aprocitentan is physiologically plausible as a testable volume-mitigation strategy to reduce proximal sodium/volume retention and thereby improve the balance between efficacy (UACR and BP reduction) and risk (edema/volume expansion); at present, however, this should be framed as a hypothesis-informed co-therapy rather than an established aprocitentan treatment standard [9, 12, 14, 15, 17]. The same conceptual framework may also support pairing endothelin blockade with aldosterone-targeting therapy in selected patients with resistant hypertension. Because CKD-associated resistant hypertension is frequently volume- and aldosterone-dominant, mineralocorticoid receptor antagonists—and, prospectively, other aldosterone-targeting approaches such as aldosterone synthase inhibition (ASI)—may represent complementary rather than competing partners to aprocitentan; however, in the absence of dedicated combination trials, this should presently be framed as a mechanistically appealing but unproven strategy rather than an evidence-based aprocitentan combination standard [1, 3, 4, 6, 16]. SGLT2 inhibition promotes mild natriuresis and osmotic diuresis with preferential decongestion, while delivering proven kidney protection across diverse CKD populations [17–19]. Relevant class-level support comes from ZENITH-CKD, in which zibotentan plus dapagliflozin achieved greater UACR reduction than dapagliflozin alone, while fluid-retention risk appeared attenuated across exposure levels and events remained dose-dependent and manageable [12, 20]. Accordingly, future trials in resistant hypertension with CKD should evaluate aprocitentan on top of contemporary standard-of-care antihypertensive therapy, including SGLT2 inhibition where indicated, with prespecified volume-management algorithms and objective congestion endpoints to identify the patients most likely to derive favorable net clinical benefit [10, 12–14, 20].

Finally, the relevance of endothelin-targeted therapy may extend to Black patients with resistant hypertension, a high-risk subgroup in whom endothelin-1 biology may be particularly relevant. In a preplanned PRECISION subgroup analysis, aprocitentan reduced BP and albuminuria in Black individuals, with BP-lowering efficacy similar to that observed in the overall trial population; peripheral edema was the most frequent adverse event, but overall tolerability remained acceptable. Although these findings should not be overgeneralized beyond the studied subgroup, they strengthen the external clinical plausibility of aprocitentan across high-risk resistant-hypertension phenotypes and support dedicated prospective evaluation in CKD-enriched Black populations [21].

## Data Availability

No new data were generated or analyzed in this study. Data sharing is not applicable to this article.
